# Amelioration of Colitis in Mouse Model by Exploring Antioxidative Potentials of an Indigenous Probiotic Strain of *Lactobacillus fermentum* Lf1

**DOI:** 10.1155/2014/206732

**Published:** 2014-07-01

**Authors:** Ritu Chauhan, Aparna Sudhakaran Vasanthakumari, Harsh Panwar, Rashmi H. Mallapa, Raj Kumar Duary, Virender Kumar Batish, Sunita Grover

**Affiliations:** ^1^Molecular Biology Unit, Department of Dairy Microbiology, National Dairy Research Institute, Karnal, Haryana 132001, India; ^2^Department of Food Engineering and Technology, Tezpur University, Napaam, Assam 784028, India

## Abstract

Based on the preliminary screening of eight indigenous putative probiotic *Lactobacilli*, *Lactobacillus fermentum* Lf1 was selected for assessing its antioxidative efficacy in DSS colitis mouse model based on its ability to enhance the expression of “*Nrf*2” by 6.43-fold and malondialdehyde (MDA) inhibition by 78.1  ±  0.24% in HT-29 cells under H_2_O_2_ stress. The Disease Activity Index and histological scores of Lf1-treated mice were lower than the control group. However, expression of “*Nrf*2” was not observed in Lf1-treated mice. A significant increase in the expression of antioxidative enzymes such as *SOD*2 and *TrxR*-1 was recorded in both of the groups. The expression of *SOD*2 was significantly downregulated in colitis-induced mice by −100.00-fold relative to control group, and the downregulation was considerably reduced to −37.04-fold in colitis Lf1 treatment group. Almost, a similar trend was recorded in case of “thioredoxin” expression, though “CAT” was refractile to expression. The Lf1-treated group had decreased malondialdehyde level as compared to colitis control (37.92  ±  6.31 versus 91.13  ±  5.76 *μ*M/g). These results point towards Lf1-induced activation of the antioxidant enzyme system in the mouse model and its prospects to be explored as a new strategy for IBD management.

## 1. Introduction

Inflammatory bowel diseases (IBDs) including Crohn's disease (CD) and ulcerative colitis (UC) have been recognized as the chronic inflammatory disorders of the gastrointestinal tract whose pathogenesis is not completely understood [1]. However, high-grade oxidative stress induced as a result of generation of excessive level of reactive oxygen species (ROS) has been commonly implicated in the pathogenesis of some chronic human disorders including autoimmune diseases and also happens to be the hall mark of IBDs [2–4]. The production of free radicals at high levels in the gut can exert cytotoxic effects on the membrane phospholipids of the intestinal epithelial cells, resulting in the formation of toxic products such as malondialdehyde (MDA). Similarly, the occurrence of severe peroxidative changes due to lipids and free radicals reaction resulting in enhanced lipid peroxidation has been found to be commonly associated with the onset of IBDs [5]. However, human body has evolved genetic programmes through intervention of antioxidative enzymes to protect itself from such oxidative stresses by maintaining cellular homeostasis and function. The key antioxidant enzymes which include superoxide dismutase (“*SOD*”), glutathione peroxidase (“*GPx*”), and catalase (“*CAT*”) form the backbone of the enzymatic antioxidant cascade and offer protection to cells and tissues against oxidative injury [6]. In this context, nuclear factor erythroid 2-related factor 2 (“*Nrf*2”) has been recognized as one of the key transcriptional factors that can play a significant protective role by controlling the antioxidant response element- (ARE-) dependent gene regulation in response to oxidative stress [7–9].

Recently, food supplementation with antioxidants has been the major focus of attention amongst the health professionals across the world to explore it as a strategy to protect against the injurious effects of oxidative stress. One of the dietary based strategies currently in vogue explores probiotics for amelioration of oxidative stress-related diseases by augmentation of antioxidant defense systems operating in the human body. Probiotics have recently emerged as the powerful microbial tools for novel therapeutic applications specifically targeted against IBD by virtue of expressing several physiologically important functions under both* in vitro* and* in vivo* conditions. Besides displaying a plethora of novel health promoting functions which are highly strain specific, probiotic bacteria including* Lactobacilli *also demonstrated strong antioxidative potentials as reported previously [11–13]. In this context, one such study merits special attention wherein* L. fermentum* ME-3, a potential probiotic strain of proven efficacy, possesses antagonistic and antioxidative properties, when used in conjunction with ofloxacin decreased lipid peroxide value in the mucosa of the small intestine and liver of* Salmonella typhimurium* infected murine model [14, 15]. As probiotics showed a positive functionality on oxidative stress-related indices, they can help both to stabilize and to promote the potency of the whole body antioxidative defense system and thus in turn may have an impact on lowering the risk of several inflammatory metabolic disorders including IBDs. Besides this, a large body of evidence also suggests that probiotics, like VSL#3 and* L. rhamnosus* GG, could serve as the promising candidates for the prevention and control of IBD although some conflicting results have also been reported [16–20].

Several animal models of IBD have been designed and amongst them dextran sulfate sodium (DSS) induced colitis model appears to represent the most accurate model of IBD since it provides human IBD-like symptoms [21]. Data from animal models of colitis have indicated that specific probiotic* Lactobacillus* and* Bifidobacterium* strains could prevent and treat intestinal inflammation [22–25]. This study was specifically undertaken to showcase the antioxidative potentials of* L. fermentum* Lf1, a promising indigenous probiotic* Lactobacillus *strain, to manage oxidative stress through “*Nrf*2” activation and modulation of lipid peroxidation by impacting MDA level under both* in vitro* (HT-29) and* in vivo* conditions in DSS colitis mouse model.

## 2. Materials and Methods

### 2.1. Ethical Statement

Before setting up the animal study in colitis mouse model, prior approval of the Institute's Animal Ethics Committee (IAEC) of the National Dairy Research Institute (NDRI, Karnal, India) was obtained. The experimental animals used in this study were maintained as per National Institute of Nutrition (NIN), India, guidelines for the care and use of laboratory animals (date of approval: 30/10/10). Surgery was performed at anesthesia conditions to minimize suffering of the animals.

### 2.2. Bacterial Strains

A total of ten bacterial cultures which included eight indigenous* Lactobacillus* isolates comprising of seven strains of* Lactobacillus plantarum* (*L. plantarum* 9, 10, 42, 55, 78, 91, and S3) and a strain of* Lactobacillus fermentum* (Lf1; an indigenous isolate of Indian gut origin, deposited in International Depository Budapest Treaty at Microbial Type Culture Collection; MTCC 5689) besides two reference probiotic strains* L. plantarum* CSCC5276 (also designated as CSCC5276, NCDO82, or VTTE-71034) which was procured from Dr. N. P. Shah from Victoria University, Australia [26, 27], and* L. acidophilus* NCFM (also known as* Howaru Dophilus*, LA-1, NCK56, NCK45, N2, RL8KR, RL8KS, and RL8K) [28–30] formed the subject of this study. They were procured from the probiotic culture collection maintained at Molecular Biology Unit, Dairy Microbiology Division, National Dairy Research Institute, Karnal. Bacterial strains were activated prior to use by subculturing in sterile de Man Rogosa Sharpe (MRS) broth (HiMedia, India) at 37°C for 18–24 h.

### 2.3. Propagation of Human Colonic Epithelial Cell Lines (HT-29 Cells) and Treatments

Human colonic epithelial cell line (HT-29) was procured from National Centre of Cell Sciences (NCCS, Pune, India). The cell line was cultured in Dulbecco's Modified Eagles Medium (DMEM, Sigma, USA). The propagation of HT-29 cells was done as reported in a previous study and carried out in our lab [31]. After attaining confluency, HT-29 cells were challenged with each of the 1 mL of live probiotic strains (~1 × 10^9^ cfu/mL resuspended in DMEM) and incubated at 37°C in 5% CO_2_ for 4 h followed by addition of fresh medium without antibiotic and foetal bovine serum. After incubation, cells were challenged with 1 mM H_2_O_2_ (Sigma, USA) and again incubated for additional 30 min. After incubation, H_2_O_2_ was completely removed and trypsinization (with Trypsin-EDTA solution; Sigma, USA) was done. Cells were again resuspended in DMEM medium and pelleted (centrifugation at 1700 rpm for 5 min at RT) for RNA isolation. The supernatant was stored at −80°C for total antioxidant activity and lipid peroxidation determination.

#### 2.3.1. “*Nrf*2” Gene Expression Study in HT-29 Cells

RNA was isolated from the cell pellet by TRIzol method (TRI Reagent, Sigma, USA) and cDNA was prepared using ImProm-II reverse transcriptase kit (Promega, USA) and random hexamer primers (100 *μ*M) provided with the same as described previously [32]. The expression of target genes “*Nrf*2” along with housekeeping gene “*β*-actin” for HT-29 cells and from HT-29 cells (after H_2_O_2_ challenge) was studied by reverse transcriptase-quantitative PCR (RT-qPCR). Untreated HT-29 cells grown in DMEM served as control. The primers used in this study are listed in Table 1.

#### 2.3.2. Lipid Peroxidation

The extent of lipid peroxidation, an index of oxidative stress was measured as thiobarbituric acid reactive substances (TBARS) formed. Lipid hydroperoxides were measured by standard TBA test method [33].

### 2.4. Experimental Design of an* In Vivo* Study Using DSS Colitis Mouse Model

A total of thirty-two adult male (seven-to-eight weeks old) Swiss Albino mice weighing 25–30 g on average were used in this study. The animals were fed normal diet (Bengal gram crushed: 58%; wheat starch: 15%; groundnut cake: 10%; casein: 4%; groundnut oil: 4%; salt mixture: 4%; vitamin mixture: 0.2%; and choline chloride: 0.2%) and water* ad libitum* during the entire course of the experiment. The animals were divided into four homogeneous groups (noncolitis control, NC-PBS; noncolitis (Lf1) control, NC-Lf1; colitis control, C-PBS; and colitis-Lf1 (C-Lf1) treatment groups) comprising of eight animals each, housed in individual cages and maintained under a constant 12 h light-12 h dark cycle. The temperature was controlled at 22–25°C with about 56–60% relative humidity. There was no significant difference in the body weight of mice among the four groups on day zero. The body weight of mice in each group on the first day was taken as the basal level. The subsequent changes in their body weight were monitored periodically during the entire 13-day experimental period. The body weight of mice each day minus the basal body weight was expressed as the body weight change. The negative value indicated the decreased weight, while the positive value represented the increased weight.

### 2.5. Feeding of Mice and Experimental Design

The mice were fed for twelve days by oral intubation with 1.5 mL of overnight grown* Lactobacillus* culture (Lf1) at 10^9^ cfu/mL after centrifugation at 7,000 rpm for 10 min and resuspending the cell pellet in 200 *μ*L of sterile PBS solution with the help of 1 mL syringe and silicon tube. The mice fed with PBS alone served as the control group. The experimental design has been illustrated in Figure 1(S) in Supplementary Material available online at http://dx.doi.org/10.1155/2014/206732. Noncolitis control group was treated with PBS alone for 12 days. Noncolitis (Lf1) treatment group was administered with 200 *μ*L* Lactobacillus* culture (Lf1) orally by orogastric tube. Colitis (Lf1) mice were fed with 200 *μ*L* Lactobacillus* culture (10^8^-10^9^ cfu/ml) orally by orogastric tube for 7 days before starting dextran sodium sulfate (DSS) and continued till 12th day after DSS induction. PBS was administered in colitis control group. Colitis was induced by 5% (w/v) DSS (MW = 40,000–50,000; USB Corporation, Cleveland, ICN Biomedicals, OH, USA) dissolved in drinking water after 7 days. All the animals in different groups were monitored on every alternate day for physical parameters like weight-loss/gain, faecal matter consistency, and blood in stools. Faecal samples were collected every three days and plated on MRS media to determine* Lactobacillus *counts in faeces as well as to assess the persistence of the viable probiotic cells in mice gut. Disease Activity Index (DAI) scores were also recorded every alternate day. Length of colon was measured and histological scores were assessed on the 13th day. Colon tissue was used for performing lipid peroxidation assay as well as for RNA isolation for gene expression studies with regard to “*Nrf*2” and antioxidant genes by RT-qPCR.

### 2.6. Evaluation of Colonic Damage and Inflammation

Severity of colitis was assessed every alternate day using a Disease Activity Index (DAI). DAI is known to be a valid measure for the acute colitis produced by DSS [34]. The scores were assigned on 0–4 scale taking into account weight loss, stool consistency, and blood in stool on scales of 0–4 (Table 1(S) in Supplementary Material) [35]. For each mouse, a daily DAI score was calculated as the aggregate of the % weight loss score, stool consistency score, and rectal bleeding divided by three. The DAI values for the groups were then recorded as mean ± standard deviation for each day of the experiment. Subsequently, the daily DAI score for each experimental group was calculated as the mean ± standard error of the individual DAI scores of each mouse in the group (Table 1(S)). The DAI clinical parameters used here are comprehensive functional measures that are somewhat analogous to clinical symptoms observed in human IBD and the scoring method that has been validated by repeated studies. Besides this, weight of mice was recorded at every alternate day as it is also correlated with severity of induced colitis. Scoring system for the comparative analysis of intestinal bleeding is given in the form of Table 1(S).

The animals were sacrificed on the thirteenth day. By carefully opening the mouse by a ventral midline incision, the colon was collected from the colon-cecal junction to the anus and its length was measured. After removal of faecal matter, colon was rinsed with DEPC water. A small part (0.2-0.3 cm) of colon was kept in 10% buffered formalin for histopathological examination (i.e., Hematoxylin and Eosin staining), and the remaining tissue was immediately wrapped in aluminium foil and snap frozen in liquid nitrogen for RNA isolation and lipid peroxidation analysis. Colonic damage and inflammation were recorded by the intensity of inflammation monitored through histopathological examination. Persistence of probiotic* Lactobacilli *was also monitored by taking faecal counts.

### 2.7. RAPD Profiles of* Lactobacillus *Faecal Isolates

Faecal samples were collected every alternate day of probiotic administration/PBS (control group). Faecal material (100 mg) was homogenized in one mL of PBS. Serial dilutions were made and plating for* Lactobacillus *counts was done on MRS-BCP agar. The plates were incubated at 37°C for 24–48 h. Colonies were counted and recorded. Colonies from different faecal count plates were randomly picked and grown for 16 to 18 h at 37°C after inoculation into MRS broth. The genomic DNA from faecal isolates was then isolated by Pospiech and Neumann method [36]. PCR with random oligo primer named 275 (5′-ccg ggc aag c-3′) was carried out for RAPD profiling. PCR reaction was performed in 25 *μ*L reaction volume containing PCR buffer (2.5 *μ*L), oligo primer (2.0 *μ*L), 200 *μ*M dNTP (2.0 *μ*L), 1.0 U Taq DNA polymerase (0.5 *μ*L), and template (2.0 *μ*L). The PCR cycling parameters included an initial denaturation of 95°C/5 min, followed by 45 cycles each of denaturation (95°C/30 sec), annealing (40°C/30 sec), extension (72°C/2 min), and final extension of 72°C/10 min. The PCR products were electrophoresed on 1.8–2% agarose gel with ethidium bromide (0.5 *μ*g/mL) (Amersham Biosciences, USA) at 100 V using 1X TAE buffer. The RAPD patterns were compared with the patterns of the pure cultures of the administered probiotic strain.

### 2.8. Histopathological Examination

The intestinal tissues from the colon of the probiotic and control groups were set apart for histopathological examination. Intestines (0.2-0.3 cm of tissue) of different groups were kept in 10% buffered formalin solution for at least 24 h prior to cassetting. The colon was sliced, mounted, stained with hematoxylin (HiMedia, India) and eosin (HiMedia, India), and then scored by a blinded pathologist who rated the tissues based on the grade of the disease, 0–4, the percentage of the disease, 0–4, and the severity of the inflammation, 0–3 (Table 2(S), a–c) [35]. Histopathological examination of the affected tissues from the mice groups analyzed in this study was done by a blinded histologist, who rated the tissues based on the grade of the disease, 0–4; the percentage of the disease, 0–4; and the severity of the inflammation, 0–3. Microscopic evaluation of all the sections of tissue samples was done at 4x and 10x magnification and histological scores were graded on the basis of the severity of inflammation, infiltration, ulceration, and crypt damage.

### 2.9. Lipid Peroxidation

For detection of lipid peroxides (malondialdehyde) in colon homogenate, the method of Uchiyama and Mihara was followed [37]. The colon homogenate was prepared in 1X PBS. An aliquot of 3 mL of 1% phosphoric acid and one mL of 0.6% TBA solution were added to 0.5 mL of colon homogenate. The mixture was heated for 45 min on a boiling water bath. After cooling, 4 mL of n-butanol was added and mixed vigorously. The butanol phase was separated by centrifugation at 5000 rpm for 10 min and absorbance was measured at 535 and 520 nm. The difference was used as the TBA value. A standard curve of malondialdehyde was drawn by taking TBA value at various concentration of malondialdehyde. MDA standard curve was prepared by carrying out overnight digestion of different concentrations of 1,1,3,3-tetraethoxypropane (0.1 mM) in presence of 0.2 N HCl.

### 2.10. Gene Expression Studies

RNA was isolated from mouse colon by TRIzol method (TRI Reagent Sigma, USA), cDNA was synthesized, and RT-qPCR in LightCycler 480 (Roche, Switzerland) was carried out as described previously [31]. The thermal cycling conditions included initial denaturation at 95°C for 5 min, followed by 40 cycles each of denaturation (95°C/30 s), annealing (53°C/30 s), and extension (72°C/45 s) with a single fluorescence measurement, a melt curve program (60°C–95°C with a heating rate of 0.11°C/s and continuous fluorescence measurement), and, finally, a cooling step at 40°C. RNA extraction and measurement of gene expression by RT-qPCR were performed in triplicate, and the mean of all these values was used for final analysis.

The quantitative data generated by real-time PCR (the quantification cycle; Cq value) were analyzed by relative expression software tool (REST 2009) (http://www.gene-quantification.info/) as described previously [32].

### 2.11. Statistical Analyses

The data obtained from each animal experiment were expressed as mean ± standard deviation values. Analyses of variance (ANOVA) for estimation of significance of differences between means were automatically done with the Statistical Package for the Social Science (SPSS for Windows, version 10.1, SPSS Inc., Chicago) software by Bonferroni post hoc test and considered significantly different at *P* ≤ 0.05.

## 3. Results

### 3.1. Screening of Probiotic Lactobacilli

**Figure 1:**
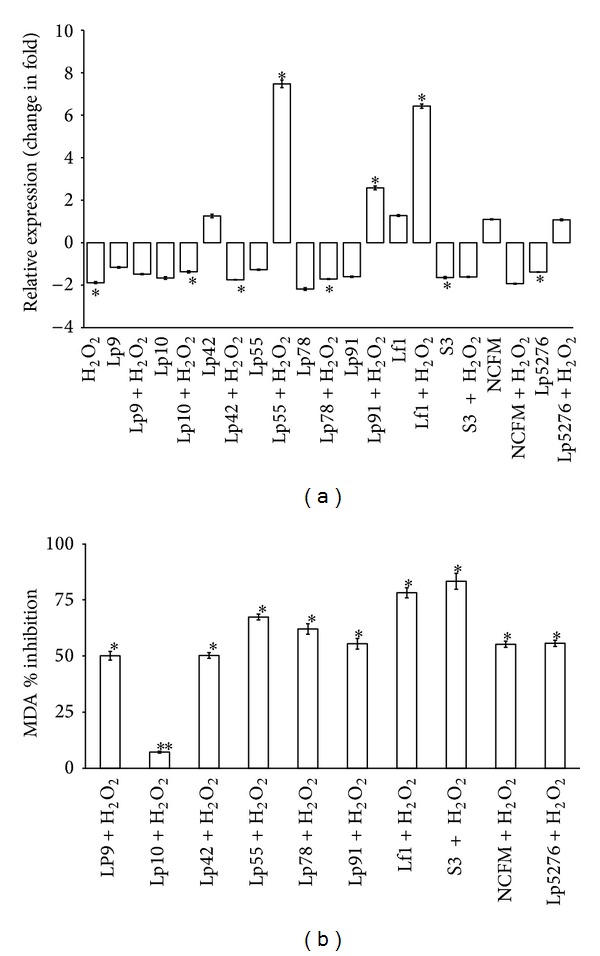
Relative expression of “*Nrf*2” and MDA inhibitory effect by* Lactobacilli* strains in HT-29 cell lines under H_2_O_2_ stress conditions. Data are represented as mean ± SD; number of RT-qPCR experiments (*n*) = 3. (a) Comparative analysis of relative expression of “*Nrf*2” in HT-29 on challenge with different strains of probiotic* Lactobacilli *and H_2_O_2_ stress. *Data are significantly different compared with control (*P* < 0.001; Bonferroni post hoc test). (b) Inhibitory effect of various probiotic* Lactobacillus* cultures on lipid peroxidation in HT-29 cells. Data are significantly different compared with control (H_2_O_2_ alone) (*P < 0.001; **P < 0.01; Bonferroni post hoc test).

Initially, during this part of study, some putative probiotic* Lactobacilli* were screened on the basis of their ability to enhance “*Nrf*2” expression in HT-29 cells along with lipid peroxidation inhibitory potential to short list the most promising strain for further investigations to explore its* in vivo* efficacy in colitis mice model. From the critical appraisal of the data recorded in Figure 1(a), it can be inferred that there was considerable variation in “*Nrf*2” expression induced in HT-29 cells by different probiotic strains as they behaved differently in eliciting “*Nrf*2” expression. The expression of “*Nrf*2” in HT-29 cells was significantly downregulated (*P* < 0.001) in the sample group (relative to control group) by −1.88-fold when subjected to H_2_O_2_ stress alone (Figure 1(a)). Although some of the probiotic strains when used alone or in conjunction with H_2_O_2_ did not show any significant effect on the “*Nrf*2” expression in HT-29 cells, Lp5276 and S3 did induce significant downregulation at the level of −1.38- and −1.64-fold, respectively. However, it was quite interesting to note that three of the strains, namely, Lp55, Lp91, and Lf1, were able to induce a significant (*P* < 0.001) upregulation in the expression of “*Nrf*2” in HT-29 cells at the levels of 7.48-, 2.58-, and 6.43-fold when used in conjunction with H_2_O_2_. Hence, the latter were rated as the prospective candidate probiotic strains to explore their efficacy in the management of oxidative stress induced diseases.

The total antioxidative activity of the aforesaid* Lactobacillus *strains in terms of their lipid peroxidation inhibition potentials in HT-29 cells was also investigated after subjecting the same to H_2_O_2_ treatment by measuring the level of MDA, the secondary product of lipid peroxidation. From the data presented in the form of a bar diagram illustrated in Figure 1(b), it is clearly indicated that* Lactobacillus* spp. S3 showed the highest percent inhibition, that is, 83.2 ± 0.53, followed by Lf1 (78.1 ± 0.24) as compared to the standard reference strains showing relatively poor response as can be reflected from the lower percent inhibition values, that is, 55.6 ± 0.4 (Lp5276) to 55.2 ± 0.36 (LaNCFM), indicating that the indigenous probiotic strains were better equipped to neutralize the injurious effects of oxidative stress. Hence, based on the overall combined effects of both of these activities in HT-29 cells, we finally selected Lf1 as the potential candidate strain for* in vivo* study.

### 3.2. *In Vivo* Studies in DSS Colitis Mouse Model

Feeding mice with DSS via drinking water for five consecutive days led to acute colitis characterized typically by bloody diarrhoea, ulcerations, and infiltrations with granulocytes. Furthermore, since DSS happens to be toxic to gut epithelial cells of the basal crypts and hence can impact the integrity of the mucosal barrier, all the experimental animals belonging to either the control or the treatment groups were critically examined for the following clinical parameters besides other important physicochemical changes recorded in mice groups during the course of this experiment.

#### 3.2.1. Weight Loss

**Figure 2:**
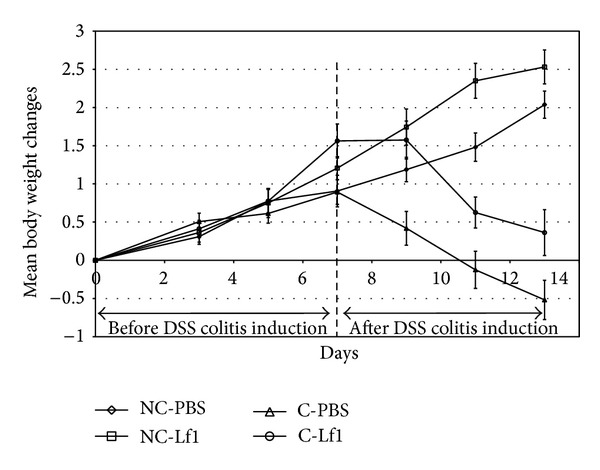
Body weight changes among different groups of mice. NC-PBS: noncolitis control; NC-Lf1: noncolitis (Lf1) control; C-PBS: colitis control; and C-Lf1: colitis-Lf1 treatment groups. The data for different groups were reported as mean ± standard deviation.

The gross changes in the body weight of mice in different treatment groups including probiotic interventions were monitored during the entire 13-day experimental period of the study. The body weight was found to increase gradually in both the noncolitis control and Lf1 groups. Mice in the colitis group showed a gradual increase in weight for the first seven days but later after starting DSS treatment excessive weight loss was recorded that became highly significant starting from day eight to day thirteen (*P* < 0.05) due to severe experimental colitis. The colitis-Lf1 treatment group showed a mixed response as there was gradual increase in body weight in the first seven days but after induction of colitis significant inhibition of weight loss was recorded (*P* < 0.05). Quite interestingly, some of the mice in the same treatment group showed the opposite effect by recording appreciable weight gain. The changes recorded with regard to body weight in different mice groups during the course of this study have been illustrated in Figure 2. However, there was no significant difference in weight gain between both NC-PBS versus NC-Lf1 and C-PBS versus C-Lf1 groups (*P* > 0.05).

#### 3.2.2. Length of Colon

The length of mice colon in the noncolitis control with respect to noncolitis Lf1 fed groups was 7.05 ± 0.21 and 8.15 ± 0.14, respectively. The length of colon of colitis-Lf1 treatment mice was much longer than that of mice in the colitis group (7.93 ± 0.38 versus 6.38 ± 0.39 cm, *P* < 0.05). There was no significant difference (*P* > 0.05) between the length of colon of colitis-Lf1 treatment group and noncolitis Lf1-treated group.

#### 3.2.3. Disease Activity Index (DAI) Scores

**Figure 3:**
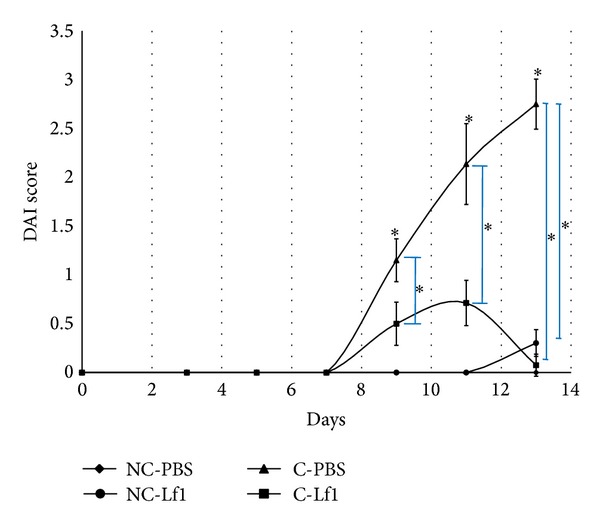
Comparative analysis of mean DAI scores in different groups in mice. NC-PBS: noncolitis control; NC-Lf1: noncolitis (Lf1) control; C-PBS: colitis control; and C-Lf1: colitis-Lf1 treatment groups. Data showing asterisk mark (∗) without line shows significant difference compared with noncolitis control (NC-PBS) (Bonferroni post hoc test); *P < 0.001. The data for different groups were reported as mean ± standard deviation.

As can be reflected from Figure 3, the highest DAI score was recorded in colitis control group. DAI scores of noncolitis control group and noncolitis probiotic group were, however, zero as weight loss changes observed in these groups were not significant to be considered as DAI and hence were rated as nonsignificant (NS) groups. However, DAI scores were improved significantly in colitis-Lf1 treatment group as shown in Figure 3. On comparative evaluation, there was significant difference (*P* < 0.05) recorded between the DAI scores of colitis control and colitis-Lf1 treatment groups due to probiotic treatment in the latter (Figure 3).

#### 3.2.4. Histological Scores

**Figure 4:**
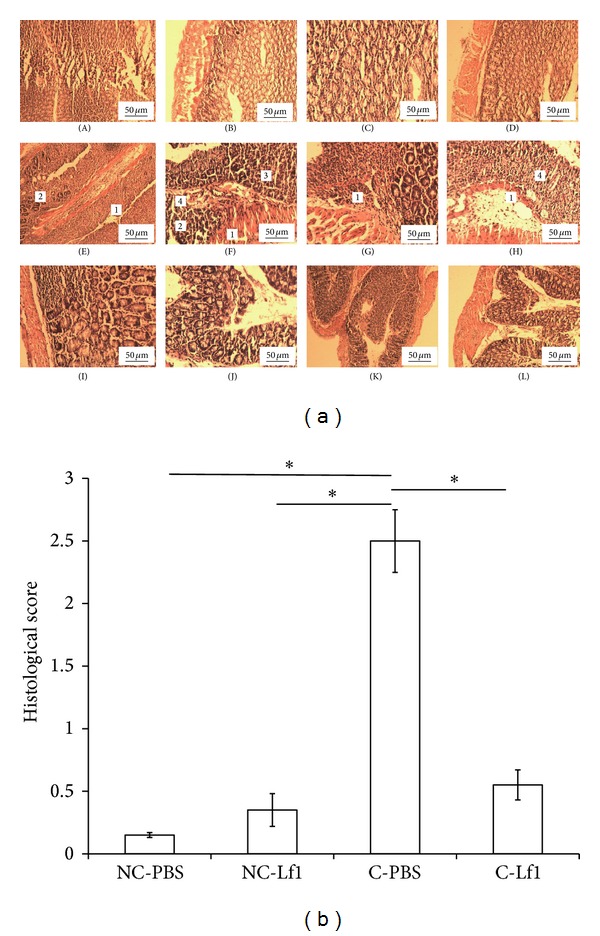
Histological examination and scores of different groups of mice. (a) Histological examination of noncolitis control group, noncolitis (Lf1) control, colitis group, and Lf1-treated group of mice. A-B: NC-PBS (noncolitis control), showing normal mucosal and epithelial layer along with intact glands with no sign of inflammation, infiltration, ulceration, and cryptitis; C-D: NC-Lf1 (noncolitis (Lf1) control), showing normal histology of noncolitis (Lf1) control similar to noncolitis control; E–H: C-PBS (colitis control), showing severity of colitis in colitis control group. (1) Inflammation infiltrate (lymphocytes and neutrophils), (2) gland destruction, (3) cryptitis, (4) loss of entire crypt with erosion of surface epithelium, and I-L: C-Lf1 (colitis-Lf1 treatment groups), showing normalization of histological score in colitis- (Lf1) treated group with no cryptitis, no ulceration, and minimal-to-moderate infiltration. (b) Histological scores/changes induced in different mice colon in different mice groups in different treatments. NC-PBS: noncolitis control; NC-Lf1: noncolitis (Lf1) control; C-PBS: colitis control; and C-Lf1: colitis-Lf1 treatment groups. *Data are significantly different within the group (*P* < 0.001, Bonferroni post hoc test). The data for different groups were reported as mean ± standard deviation.

Microscopic examination of the affected tissues from the noncolitis control group revealed normal colonic epithelium with intact crypts and glands (Figures 4(a) and 4(b)). However, a mild lymphocytic infiltration was also discernible although such minor changes were also recorded even under normal conditions of inflammatory response. Almost a similar trend was also recorded in biopsies of noncolitis Lf1 mice. On the other hand, biopsies of the colitis mice revealed focally ulcerated epithelium with moderate-to-excessive inflammatory infiltrate primarily composed of polymorphs and lymphocytes. Crypt abscesses along with their shortening and branching were also recorded accompanied by focal lymphoid follicle formation, cryptitis, and gland destruction. These symptoms gave indication that acute colitis was developed in mice. Contrary to this, biopsy studies of the colitis-Lf1 treatment group demonstrated considerable improvement in the tissue damage as can be reflected from the intact epithelium with mild, acute, and chronic inflammatory infiltration of lymphocytes. Nonetheless, focal lymphoid follicle formation, ulcerated epithelium with moderate inflammatory infiltrate composed of polymorphic lymphocytes, focal cryptitis, and gland destruction were also recorded in two of the samples in this group. Similarly, there was no evidence of granulomatous inflammation or malignancy or cryptitis or crypt abscess.

#### 3.2.5. Persistence of* L. fermentum* Lf1 in Colitis and Noncolitis Models

**Figure 5:**
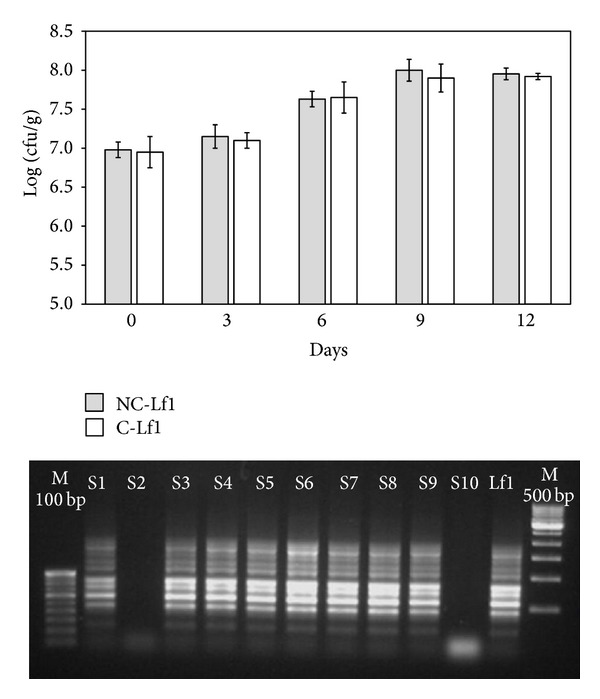
Persistence and RAPD pattern of faecal isolates and standard culture Lf1 using primer 275 in faecal samples of mouse. NC-Lf1: noncolitis (Lf1) control and C-Lf1: colitis-Lf1 treatment groups. The data for different groups were reported as mean ± standard deviation.

The persistence and viability of Lf1 in mice gut were confirmed by monitoring the log counts in the faecal samples from different mice groups and also subjecting some randomly selected colonies of faecal isolates recovered on MRS agar (Figure 5) by species specific PCR (data not shown) and RAPD to ascertain whether they belonged to Lf1 or not. From the log count data presented in Figure 5, it is quite clear that Lf1 not only persisted in the mice gut in all the treatment groups but also proliferated as can be reflected from the gradual increase in the faecal* Lactobacillus *log counts during the treatment period registering more than one log cycle increase towards the end of the experimental period. The preponderance of Lf1 in the faecal* Lactobacillus *isolates in the faecal samples of mice was demonstrated by PCR and RAPD. The typical RAPD banding patterns of pure Lf1 along with some of the randomly selected representative faecal* Lactobacillus *isolates obtained with primer 275 have been recorded in Figure 5. Since the typical RAPD banding patterns recorded with most of the faecal isolates matched those obtained from the pure culture of Lf1, it gives indication that most of the faecal isolates belonged to the clones of Lf1 that was administered to the mice. From these results, it can be inferred that Lf1 could survive and persist in the colon to express its antioxidative functions.

### 3.3. MDA Level as an Index of Lipid Peroxidation

**Figure 6:**
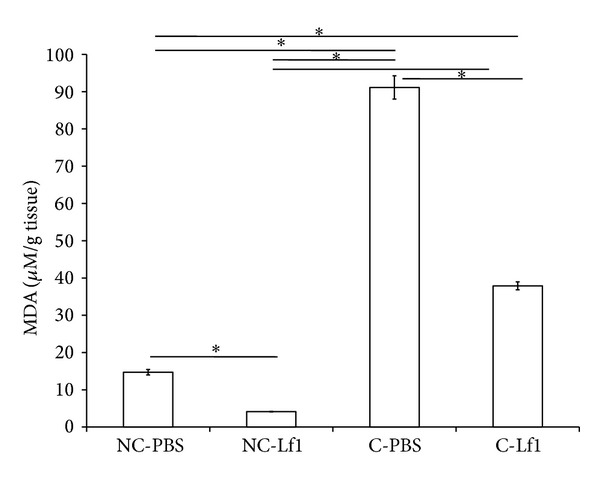
Comparative evaluation of MDA levels recorded in the colonic tissue of experimental mice group for different treatments. NC-PBS: noncolitis control; NC-Lf1: noncolitis (Lf1) control; C-PBS: colitis control; and C-Lf1: colitis-Lf1 treatment groups. *Data are significantly different within the group (*P* < 0.001, Bonferroni post hoc test). The data for different groups were reported as mean ± standard deviation.

The data on the level of MDA estimated in each tissue sample from each mice group under different treatments is recorded in Figure 6. There was significant difference in the MDA content of colitis control (91.13 *μ*M/g) and colitis-Lf1 treatment group (37.92 *μ*M/g), thereby suggesting that our probiotic strain exhibited strong antioxidative property in colon and prevented lipid peroxidation by decreasing the level of MDA at significant level.

### 3.4. Relative Expression Studies of “*Nrf*2” and Antioxidative Enzyme Systems (“Superoxide Dismutase 2”, Thioredoxin Reductase, and Catalase) in Colitis Mouse Model by RT-qPCR

**Figure 7:**
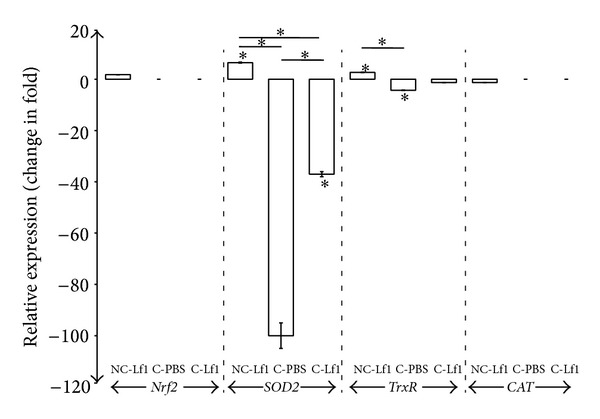
Relative expression of* Nrf*2,* SOD*2,* TrxR,* and* CAT* in colitis mouse model. NC-Lf1: noncolitis (Lf1) control; C-PBS: colitis control; and C-Lf1: colitis-Lf1 treatment groups. Data are represented as mean ± SD; number of RT-qPCR experiments (*n*) = 3. Data showing asterisk mark (∗) without line shows significant difference compared with noncolitis control (Bonferroni post hoc test); *P < 0.001.

Specificity of RT-qPCR products generated from the amplification of reference gene “*ACTB*” and the target genes “*Nrf*2,” “*SOD*2,” “*TrxR*-1,” and “*CAT*” using specific primers was checked on agarose gel. Specific single product of the desired amplicons, that is, 102 bp (“*Nrf*2”) and 112 bp (“*ACTB*”) (Figure 2(S) in Supplementary Material), resulted in a single product of specific melting temperatures of 83.10°C and 83.20°C for “*Nrf*2” and “*ACTB,*” respectively, by melt curve analysis (Figure 2(S)). However, no Cq values in amplification curve for “*Nrf*2” gene were obtained in the colitis-induced mice as well as Lf1 fed-colitis-induced mice groups. The mice fed with probiotic strain alone in noncolitis group also did not show any significant level (*P* < 0.231) of expression of “*Nrf*2” which turned out to be only 1.756 relative to control group (Figure 7). Similarly, no significant level of expression of the target gene could be demonstrated in both untreated colitis-induced mice and Lf1-treated colitis mice since no amplification of the target gene was recorded in both of the mice groups.

With respect to antioxidant enzymes systems, specific single products of the desired amplicons, that is, 87 bp (“*SOD*2”), 65 bp (“*TrxR-*1”), and 64 bp (“*CAT*”), were obtained. All the curves showed high linearity (Pearson correlation coefficient R2 > 0.978). The slopes of “*SOD*2,” “*TrxR*-1,” “*CAT*,” and “*ACTB*” curves were −3.351, −3.363, −3.349, and −3.360, respectively, which indicated corresponding high real-time PCR efficiencies of 1.988, 1.98, 1.989, and 1.984 in respect of the targeted genes (Figure 3(a)). On melt curve analysis, a single product of specific melting temperatures of 82.59, 83.53, 82.62, and 83.16°C was recorded for “*SOD*2,” “*TrxR*-1,” “*CAT*,” and “*ACTB,*” respectively (Figure 3(b)). The relative expression of “*SOD*2” in different mice treatment groups has been recorded in Figure 7. The expression of “*SOD*2” was significantly (*P* < 0.000) downregulated in colitis-induced mice at the level of –100.00-fold relative to PBS control group. However, when the colitis-induced mice group was fed with Lf1, the downregulation was considerably reduced to −37.037-fold indicating that there was actually a substantial increase (approximately 63%) in the expression of “*SOD*2” gene under these conditions. On the other hand, the mice group fed with Lf1 alone showed a significant level (*P* < 0.000) of upregulation by as much as 6.446-fold in “*SOD*2” expression.

The expression of “thioredoxin” was also found to be significantly (*P* < 0.000) downregulated in colitis-induced mice at the level of −4.292-fold with respect to noncolitis PBS control mice. However, when the colitis-induced mice were fed with Lf1, there was no significant (*P* < 0.428) relative change in expression profile with respect to control group, although the expression level decreased from −4.292-fold in the colitis group to −1.328-fold in colitis-Lf1 mice. These results indicate that downregulation of the expression was not that drastic when Lf1 was used in the colitis-induced mice group in comparison to the untreated colitis group. On the other hand, the noncolitis mice group fed with Lf1 alone resulted in a significant level (*P* < 0.031) of upregulation of “thioredoxin” gene expression to the extent of 2.656-fold. The mice group fed with Lf1 did not show any significant level (*P* < 0.493) of relative changes (−1.29) in expression profile with respect to control group. However, expression of “*CAT*” gene could not be demonstrated in other treatment groups since amplification could not be recorded in the colitis-induced mice as well as Lf1 fed-colitis-induced mice groups.

## 4. Discussion

Low-grade inflammations accompanied by acute phase oxidative stress to the affected tissues in the gut are the hallmark of IBDs. Hence, to neutralize the damaging effects of oxidative stress, antioxidant supplements are popularly consumed with the belief that loading of antioxidant capacity through external sources could improve the ability to downsize/restrict potential oxidative damage caused by reactive oxygen species (ROS). However, recent advances made in understanding redox homeostasis maintained via the “*Nrf*2” signalling pathway may challenge the concept of artificially supplying the body with antioxidants. In this situation, the feedback nature of the redox system must be considered fully as too much ingestion of antioxidants may actually diminish the body's endogenous defensive antioxidative capability with serious health implications. Hence, the better and safer option to deal with the severe oxidative stress induced damage in the gut would be by exploring antioxidative potentials of probiotic bacteria since excessive antioxidative response coming from the probiotic strains may be downregulated by the stabilizing systems operating in the whole organisms, thereby suggesting the feasibility of the probiotic therapy to manage IBD from a broader perspective. The major focus of this study centred on “*Nrf*2” signalling pathway and its modulation by probiotic strains under both* in vitro* and* in vivo* conditions. Another important issue that figured prominently in the study was to find whether the antioxidative efficacy of Lf1 driven by “*Nrf*2” signalling pathway that was successfully demonstrated in HT-29 cells could be replicated in colitis mice model or not. This was considered important since the rationale for selecting Lf1 for demonstrating its efficacy in* in vivo* studies in colitis mice model in this investigation was based on its* in vitro* “*Nrf*2” modulatory activity and also by virtue of showing high antioxidative activity as reported previously [13].

The outcome of this study indicated that* L. fermentum* Lf1 was quite effective in reducing the severity of colitis in mice by bringing about significant changes in DAI score, body weight gain or loss, colon length, faecal character, and faecal bleeding (bloody stool) besides histological damage severity scores. A drastic weight loss in mice was recorded that became highly significant starting from the eighth day to the thirteenth day (*P* < 0.05) due to severe experimental colitis. However, significant difference was recorded in weight gain in colitis control versus colitis-Lf1 treatment groups. Our observations in this regard are in accordance with earlier reports, stating weight gain in probiotics-treated group [38, 39]. The probiotic-treated group of mice had longer colon relative to colitis control as well as noncolitis control groups [39]. The shortening of the colon under severe colitis conditions might be attributed to thickening of the colon epithelial wall that could bring about contraction of the colon length. DAI scores in Lf1 treatment group decreased gradually and reached as low as zero on the 13th day in some mice. Significant differences (*P* < 0.05) in the DAI scores of treatment and colitis control groups demonstrated the efficacy of Lf1 against colitis which is consistent with the similar observations in DAI results recorded by several other investigators [38–40]. Significant differences in histological scores amongst the colitis control and treatment groups were also recorded, which implies that Lf1 was effective in preventing histological damage of treated mice to a significant level (*P* < 0.05) which is perfectly in line with those of earlier reports on improvement in histological damage in colitis mice fed with probiotics [38–40].

The significant differences in the MDA content in the colitis control versus the colitis-Lf1 treatment group as shown in our study suggest that Lf1 exhibited a strong antioxidative property in colon as well and prevented lipid peroxidation. These findings are in concordance with that of Ito et al., who demonstrated the highest inhibitory activity against lipid peroxidation in liposomes by* Streptococcus thermophilus* YIT 2001 [41]. Our findings on the antioxidative efficacy of Lf1 in terms of lipid peroxidation inhibition in colitis-Lf1 mice can also be supported by a similar study conducted in Sprague-Dawley rats previously [42] wherein different probiotic and prebiotic based treatments alone or in combination prior to colitis induction with DSS were also shown to evoke a significant decrease in the levels of lipid peroxidation in probiotic group. Similarly, two additional studies carried out by Singh et al. [43] and Kaushal and Kansal [44] demonstrated antioxidative efficacy of low-fat probiotic dahi prepared with regular dahi cultures plus* L. acidophilus* and* L. casei* as well as* B. bifidum* by significantly reducing the levels of TBARS in colon carcinogenesis in rats/mice in comparison to DMH control rats [43]. These observations further substantiate the findings of our study and reaffirmed the antioxidative efficacy of Lf1 strain against colitis.

### 4.1. Relative Expression Studies

Since the selection of Lf1 was based on its ability to induce “*Nrf*2” expression in HT-29 cells, the expression of the same was also investigated under* in vivo* conditions using colitis model. This was considered important because* Nrf*2/ARE signalling pathway plays a crucial role in vascular homeostasis and the defence of endothelial and gut epithelial cells against oxidative stress encountered during inflammatory process. However, surprisingly, Lf1 could not induce “*Nrf*2” expression in the mice model. This was quite unexpected and inconsistent with the response recorded in HT-29 cells. Although the exact cause of differential “*Nrf*2” expression under* in vitro* and* in vivo* conditions cannot be explained precisely due to lack of any supportive data, it might be attributed to some inhibitory factor present in the complex mice gut milieu that could interfere with the processing and nuclear translocation of functional “*Nrf*2.” Hence, it would be interesting to identify such unknown factors that eventually may provide a clue to explain this hypothesis. Our findings in this regard, however, are at variance with results of previous research groups, who demonstrated increased level of “*Nrf*2” expression in rat model with liver fibrosis fed with blueberry after 21 days of treatment [45, 46]. Another possible reason for the failure of “*Nrf*2” expression in colitis mice with Lf1 could be ascribed to differences in the treatment periods in the two studies and also due to different mechanisms using different animal models followed in the two studies. Furthermore, it is to be clarified that the results from the mice experiment were recorded only up to 13 days which might be inadequate to demonstrate the effect unlike the 21 days used by Wang et al. [45, 46]. Moreover, strain specificity of the probiotic cultures could also partly account for variation in the results, thereby necessitating further standardization of the experimental design to optimize the “*Nrf*2” expression conditions in the animal models. Hence, in order to showcase the exact cause of this contradiction in results under* in vitro* and* in vivo* conditions, more robust in-depth studies are needed to unravel the intricacy of this process by optimizing the experimental conditions and proper design of the animal studies. This is important from regulatory perspectives since EU has planned new restrictions and regulations for animal models.

The antioxidative potentials of Lf1 were found to be reinforced with a powerful arsenal of several enzymatic and nonenzymatic factors such as antioxidative enzymes and other important molecules which could play a protective role during the oxidative stress induced conditions confronted in the gut. Our findings on “*SOD*2” expression in the mouse model evoked with Lf1 are well supported by the outcome of some previous studies [47–49] wherein an upregulation of “*SOD*2” was also recorded with their respective probiotic food preparations. Their results showed that probiotic dahi could increase the activity of superoxide dismutase in RBCs and colorectal tissues of rats after treatment with probiotic dahi. However, our results on upregulation (6-446-fold) of “*SOD*2” in noncolitic mice when fed with Lf1 for a period of 12 days are at variance with those earlier reports on the effect of daily consumption of probiotic and conventional yoghurt on oxidant and antioxidant parameters in plasma of young healthy women [50, 51]. Both studies reported that the probiotic and the conventional groups did not have any significant difference in “*SOD*” levels before and after probiotic intervention (*P* > 0.05). This could be attributed to the use of a different probiotic strain and also the host specificity used in the two studies.

Our results with regard to upregulation (2.656) of “*TrxR-*1” in noncolitic mice when fed with Lf1 for a period of 13 days and nonsignificant effect of probiotic feeding in colitis mice group cannot be substantiated due to lack of any published reports on these lines. Nevertheless, there are few studies reporting the positive effects of upregulation of thioredoxin reductase in the alleviation of cardiac dysfunction and protection against oxidative damage brought about by chemoprotective agents such as resveratrol [52]. Almost a similar trend was recorded with regard to catalase in our study as Lf1 did not evoke any significant effect on expression of catalase and in fact there was not even basal level expression recorded in the colitic mice group. Our findings in this regard are almost similar to those of previous published report on the clinical efficacy of probiotic preparations in healthy women which reported that the mean activities of catalase decreased significantly (*P* < 0.001) in the probiotic group, after consuming yoghurt daily for four weeks [50]. Nonetheless, some previous studies show that feeding of probiotic dahi increased the activities of catalase in different tissues in their respective studies [47, 48]. Besides this, there was an interesting study carried out by Chamari et al. that reported that catalase levels in plasma of young healthy women in a randomized clinical trial in probiotic group increased significantly after probiotic intervention in comparison with baseline (*P* < 0.001) and the mean changes in catalase level were significantly different in probiotic and conventional groups (*P* < 0.001) [51].

## 5. Conclusion

From the outcome of this study, it can be concluded that our indigenous* Lactobacillus fermentum* strain Lf1 is adequately equipped with the multifactorial antioxidative and anti-inflammatory defense arsenal not only to protect its own survival but also to confer protection to the host cells against the hostile oxidative stress confronted in the mice gut during colitis. However, it remains to be seen whether the antioxidative efficacy of this strain is mediated by activation of “*Nrf*2” stress response pathway or through other routes in established animal models. Nevertheless, it has the potential to be explored as prospective functional therapeutics to manage IBDs by establishing its clinical efficacy in well-designed double-blind placebo-controlled human trials.

## Supplementary Material

The grading of Histological changes was done by the methods given previously by Meerveld and Tyler [35]. The scoring of histological changes were done as “0” for crypt intact; “1” for loss of 1/3 crypt; “2” for loss of 2/3 crypt; “3” for loss of entire crypt with intact surface epithelium; and “4” for loss of entire crypt with erosion of surface epithelium. The extend of histological damage (in % involvement) in experiment animals were scores as “0” for 1–25% damage; “1” for 26–50% damage; “2” for 51–75% damage and '3' for 76–100% damage. While, the severity of the inflammation were scored as “0” for normal appearance; “1” for focal inflammatory cell infiltrate; “2” for inflammatory cell infiltrate, gland drop out and crypt abscess; “3” for mucosal ulceration respectively.

## Figures and Tables

**Table 1:** Sequences of the primers used for RT-qPCR.

Genes	Primer sequence (5′-3′)	Amplicon size (bp)
*Nrf*2 HF *Nrf*2 HR	gcg acg gaa aga gta tga c gtt ggc aga tcc act ggt tt	181

*Nrf*2 MF *Nrf*2 MR	ttc agc aca aca ctg gga ag tgt tgc tgg ggt tta tag gc	102

*SOD*2 MF *SOD*2 MR	gct ggc ttg gct tca ata ag taa ggc ctg ttg ttc ctt gc	87

*CAT*1 MF *CAT*1 MR	gct gag aag cct aag aac gca cct tcg cag cca tgt gag a	64

*Trx*-1 MF1 *Trx*-1 MR1	tcc att tcc atc tgg ttc tgc ttc acc att ttg gct gtt gc	65

*β*-Actin F *β*-Actin R	tgg ctg ggg tgt tga agg tct agc acg gca tcg tca cca act	238 [10]

*β*-Actin mus2F *β*-Actin mus2R	agt gtg acg ttg aca tcc gta gcc aga gca gta atc tcc ttc t	112
