# Supplementary material for: Amelioration of Colitis in Mouse Model by Exploring Antioxidative Potentials of an Indigenous Probiotic Strain of Lactobacillus fermentum Lf1
Source: Biomed Res Int. 2014 Jul 1;2014:206732. doi: 10.1155/2014/206732 (PMC4100452; doi:10.1155/2014/206732)
Supplement: Supplementary file 1 — The grading of Histological changes was done by the methods given previously by Meerveld and Tyler [35]. The scoring of histological changes were done as “0” for crypt intact; “1” for loss of 1/3 crypt; “2” for loss of 2/3 crypt; “3” for loss of entire crypt with intact surface epithelium; and “4” for loss of entire crypt with erosion of surface epithelium. The extend of histological damage (in % involvement) in experiment animals were scores as “0” for 1–25% damage; “1” for 26–50% damage; “2” for 51–75% damage and '3' for 76–100% damage. While, the severity of the inflammation were scored as “0” for normal appearance; “1” for focal inflammatory cell infiltrate; “2” for inflammatory cell infiltrate, gland drop out and crypt abscess; “3” for mucosal ulceration respectively. [file 206732.f1.pdf]

Supplementary Data:

Fig. 1S Detailed description of *In vivo* experimental design

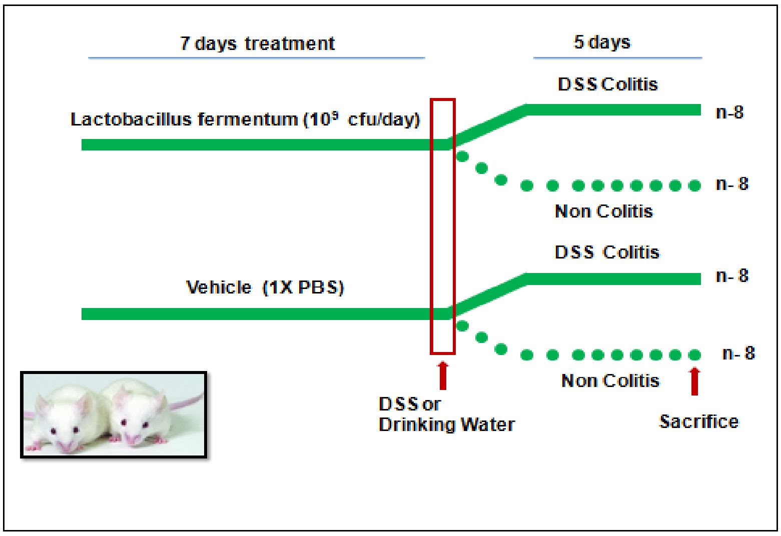

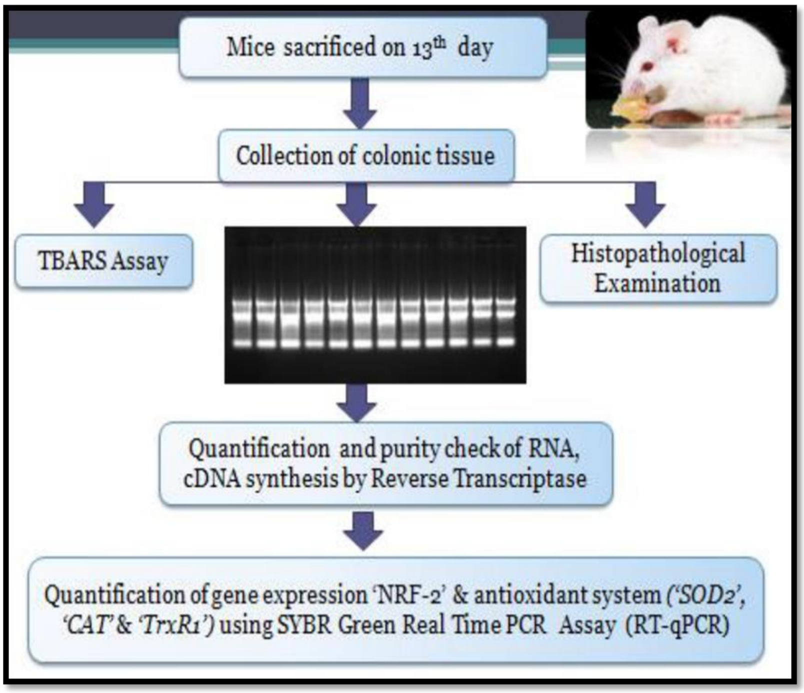

Relative Expression Studies

The primers for the house keeping genes (ACTB for mouse colon) and target genes (*Nfr*2, *SOD*2, *CAT*1 and *TrxR*-1 from mouse colon) were either taken from the published papers or designed from the genome sequence available at NCBI gene bank and using Primer 3 plus software (http://www.bioinformatics.nl/cgi-bin/primer3plus/primer3plus.cgi). The primers were got custom-synthesized from Imperial Biomed (Integrated DNA Technologies, Inc.; www. idtdna.com) and Sigma, USA. While designing the primers for Real Time PCR, the G-C content was kept within 40-60%, the amplified products size in the range of 100-200 bp and Tm in between 55-60°C. The specificity of the designed primer pair was ensured by BLAST analysis. The amplified products were checked on 2% agarose gel followed by melt curve analysis of the PCR amplified products to rule out the possibility of primer dimer formation.

i) Melt Curve Analysis and Amplification of *Nfr*2

The amplification parameters for '*Nfr*2' included initial denaturation at 95°C for 5 min. followed by 40 cycles each of denaturation at 95°C for 30 sec, annealing at 53°C for 30 sec. and extension at 72°C for 45 sec. and for '*ACTB*' were kept same as for '*Nfr2*'. Furthermore, in order to calculate RT-qPCR efficiencies and linearity, standard curves were prepared for the target and reference gene by plotting Cq values versus log cDNA (reverse transcribed total RNA) concentration inputs by preparing serial dilutions of cDNA i.e. 300, 200, 100, 50 and 12.5 and 6.25 ng/µl for '*ACTB*' and 100, 50, 25, 12.5, 5 and 1 ng/µl for '*Nfr*2' respectively. As is quite evident from the **Fig. 2S (Supplementary Data**), both the curves showed high linearity (Pearson correlation coefficient R2 > 0.978). The slopes of '*Nfr*2' and '*ACTB*' curves were -3.363 and -3.360 respectively that indicated high real-time PCR efficiencies of 1.983 and 1.984 for both the genes.

Figure: 2S Melt Curve Analysis and Amplification Curves of '*Nfr2*' and '*ACTB*'

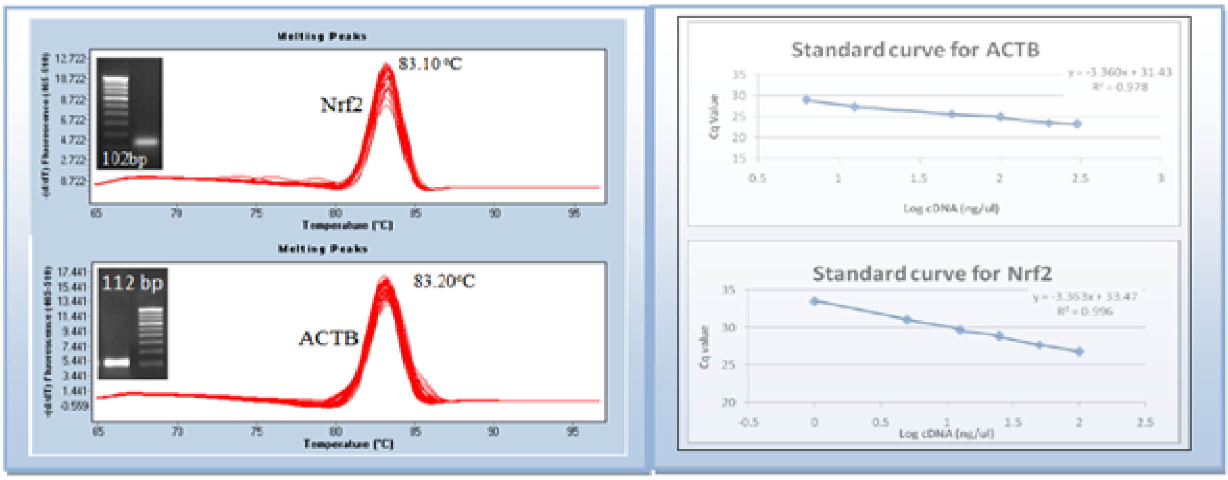

ii) Melt Curve Analysis and Amplification curve of '*SOD2'*, Thioredoxin Reductase and Catalase

'*SOD*1', '*TrxR*-1' and '*CAT'* as well as '*ACTB*' from the given slopes plotted with the help of LightCycler software. Cq values versus log cDNA (reverse transcribed total RNA) concentration inputs by preparing serial dilutions of cDNA i.e. 300, 200, 100, 50 and 12.5 and 6.25 ng/µl for '*ACTB*' and 100, 50, 25, 12.5 and 5 ng/µl target genes respectively were plotted to calculate the slope (mean ± SD; *n* = 3). The corresponding real-time PCR efficiency (*E*) of one cycle in the exponential phase was calculated according to the equation: $E = 10^{[-1/\text{slope}]}$. As is quite evident from the **Fig. 3Sa,** all the curves showed high linearity (Pearson correlation coefficient R2 > 0.978). The slopes of '*SOD*2', '*TrxR*-1', '*CAT'* and '*ACTB*' curves were -3.351, -3.363, -3.349 and -3.360 respectively that indicated high real-time PCR efficiencies of 1.988, 1.98, 1.989 and 1.984 in respect of the targeted genes. '*SOD*1', '*TrxR*-1' and '*CAT'* as well as '*ACTB*' from the given slopes plotted with the help of LightCycler software. Cq values versus log cDNA (reverse transcribed total RNA) concentration inputs by preparing serial dilutions of cDNA i.e. 300, 200, 100, 50 and 2.5 and 6.25 ng/µl for '*ACTB*' and 100, 50, 25, 12.5 and 5 ng/µl target genes respectively were plotted to calculate the slope (mean ± SD; *n* = 3). The corresponding real-time PCR efficiency (*E*) of one cycle in the exponential phase was calculated according to the equation: $E = 10^{[-1/\text{slope}].}$

Fig. 3Sa : Standard curves for the target and reference genes

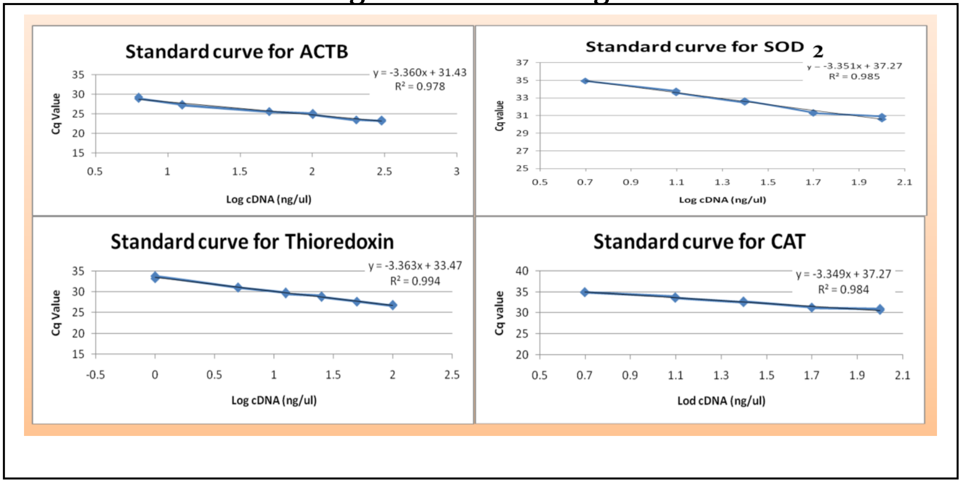

The amplification parameters for '*SOD*2' and '*ACTB'* included initial denaturation at 95°C for 5 min. followed by 40 cycles each of denaturation at 95°C for 30 sec, annealing at 53°C for 30 sec. and extension at 72°C for 45 sec

Fig. 3Sb. Specificity of RTqPCR products on agarose gel and melt curve analysis of '*SOD2'*, Thioredoxin Reductase and Catalase in colitis mouse model

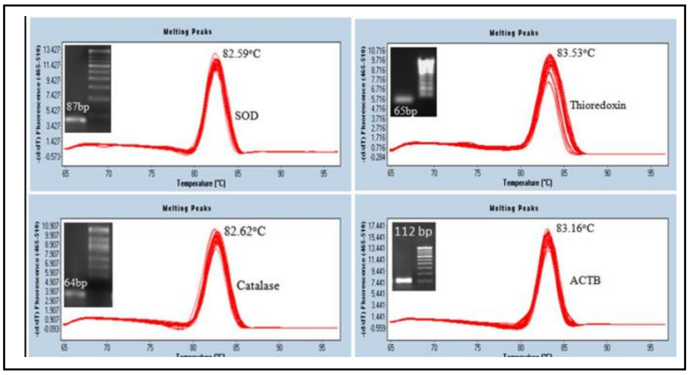

Table.1S – Disease activity index parameters

Parameter	Change	Score
Weight loss	0	0
	1 to < 5%	1
	5 to < 10%	2
	10 to < 20%	3
	>20%	4
Stool Consistency	Negative	0
	Loose	2
	Diarrhoea	4
Blood in stool	Normal	0
	Blood traces	2
	Gross Bleeding	4

Table. 2Sa -Grade of Histological Changes

Grade of Disease	Score
Crypt intact	0
Loss 1/3 crypt	1
Loss of 2/3 crypt	2
Loss of entire crypt with intact surface epithelium	3
Loss of entire crypt with erosion of surface epithelium	4

Table. 2S b – Extent of Histological damage recorded in experimental animals

Extent of Damage (% involvement)	Score
1-25%	0
26-50%	1
51-75%	2
76-100%	3

Table. 2S c – Severity of Histological damage recorded in experimental animals

Score for Severity	Score
Normal	0
Focal inflammatory cell infiltrate	1
Inflammatory cell infiltrate, gland drop out and crypt abscess	2
Mucosal ulceration	3

Source: Meeryeld and Tyler, 2006
